# Transcultural validation of the “Body Esteem Scale for Adolescents and Adults” among Tunisian adolescents

**DOI:** 10.1192/j.eurpsy.2023.1213

**Published:** 2023-07-19

**Authors:** H. Rezgui, S. Bourgou, H. Ben Youssef, R. Gadhoum, M. Hamza, S. Hadj Amor, R. Fakhfekh, A. Belhadj

**Affiliations:** 1 Child and Adolescents psychiatry, Mongi Slim Hospital; 2 Ministry of health; 3 Abderrahmen Mami Hospital, Tunis, Tunisia

## Abstract

**Introduction:**

Adolescence is considered as a particularly vulnerable period for body image disturbance. Body esteem is defined as the self-evaluation of one’s own body or appearance.

**Objectives:**

Validate the Body Esteem Scale for Adolescents and Adults (BESAA) in Tunisian adolescents.

**Methods:**

We conducted a cross-sectional study among adolescents who attend Tunisian high school from 11 October 2021 to 11 November 2021.

We translated the BESAA into dialectal Tunisian Arabic based on the translation back-translation method. The validity of the scale was evaluated through content validity, reliability and construct validity. We used the Arabic version of Rosenberg Self Esteem Scale as an external validator.

**Results:**

We recruited 340 adolescents aged between 12 and 19 years’ old. The translated version was considered satisfactory. The internal consistency showed a good result with a Cronbach Alpha of 0,830. The correlation between items and subscales demonstrated statistically significant and logical results. Statistically significant correlations were found between the BESAA and its external validator the Rosenberg Self Esteem Scale (r= 0,422; p< 0,01). The exploratory analysis related three factors similarly to the original version of the questionnaire and in confirmatory analysis. The scale demonstrated good model fit statistics as follow: Comparative fit index= 0,87; goodness of fit index=0,81; adjusted goodness of fit index=0,77; Root Mean Square Error of Approximation=0,1 and Standardized Root Mean Square Residual=0,09.

**Conclusions:**

Our BESAA version can be reliably used to conduct further studies and researches on body esteem in the Tunisian population.

**Disclosure of Interest:**

None Declared

